# A tongue-like Sinus of Valsalva Aneurysm with a pin-hole

**DOI:** 10.1093/ehjcr/ytad559

**Published:** 2023-11-09

**Authors:** Satoshi Hata, Masaoki Miyamoto, Yasushi Okumoto, Keizo Kimura

**Affiliations:** Department of Cardiology, Kinan Hospital, 46-70 Shinjyo-chou, Tanabe, Wakayama 646-8588, Japan; Department of Cardiology, Kinan Hospital, 46-70 Shinjyo-chou, Tanabe, Wakayama 646-8588, Japan; Department of Cardiology, Kinan Hospital, 46-70 Shinjyo-chou, Tanabe, Wakayama 646-8588, Japan; Department of Cardiology, Kinan Hospital, 46-70 Shinjyo-chou, Tanabe, Wakayama 646-8588, Japan

## Case description

A 66-year-old Japanese male was referred to our hospital because of a heart murmur at the 4th left sternal border detected on a health check-up. He had been diagnosed with a heart murmur in his early childhood. Still, he had never undergone a thorough medical examination since he had no heart symptoms, such as dyspnoea and leg oedema.

**Figure 1:**
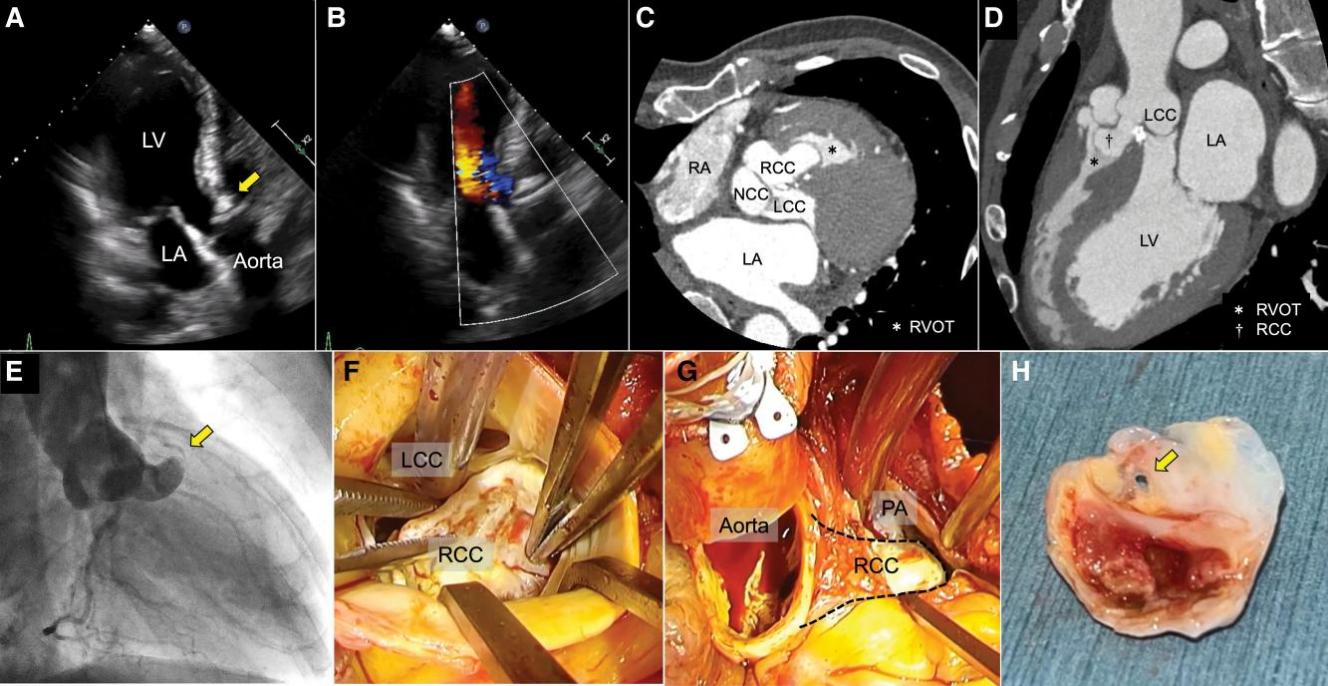
(*A* and *B*) Transthoracic echocardiography demonstrated the severe calcified right coronary cusp (arrowhead) and moderate aortic regurgitation. The diameter at the level of the sinus of Valsalva was 36 mm. Contrast-enhanced computed tomography axial view (*C*) and left anterior oblique view (*D*) showing the deformed right coronary cusp reached the right ventricular outflow tract. (*E*) Aortography demonstrated the deformed right coronary cusp with a shunt flow to the right ventricular outflow tract (arrowhead). (*F*) The aortic valve was a tricuspid, and the right coronary cusp was enlarged and calcified and had limited mobility. (*G*) The right coronary cusp was deformed, lodged into the ventricular septum, and reached the right ventricular outflow tract. (*H*) The resected right coronary cusp shows a pin-hole (arrowhead). RCC, right coronary cusp; LCC, left coronary cusp; NCC, non-coronary cusp; RA, right atrium; LA, left atrium; LV, left ventricle; RVOT, right ventricular outflow tract. ＊Right ventricular outflow tract; †right coronary cusp.

Transthoracic echocardiography (TTE) revealed a right ventricular outflow tract (RVOT) obstruction by an aneurysm originating from the right coronary sinus. The right coronary cusp (RCC) was severely calcified and enlarged, and moderate aortic regurgitation was also found (*Figure 1A* and *B*; see Supplementary material online, *S1*–*S3*). Contrast-enhanced computed tomography confirmed that the enlarged RCC extended to the RVOT (*Figure 1C* and *D*; see Supplementary material online, *Image S1*). The right-sided heart catheter test indicated the RVOT pressure gradient reached 80 mmHg without significant oxygen saturation increase in the RVOT. Aortography showed a tongue-like aneurysm with a shunt flow (*Figure 1E*; see Supplementary material online, *S4*). The value for *Q_p_*/*Q_s_* was 1.0 due to the minimal shunt volume, which did not lead to any heart symptoms.

Finally, the patient was diagnosed with a ruptured sinus of Valsalva (SoV) aneurysm. Although metastatic disease can coexist with a SoV, we could not find it. We performed surgical intervention because it is traditionally recommended for a ruptured SoV aneurysm, and the right ventricular afterload was supposed to be extremely high. The aortic valve consisted of a tricuspid. The calcified RCC was lodged into the ventricular septum at the membranous septal level and had limited mobility (*Figure 1F* and *G*). A pin-hole was located at the tip of the aneurysm (*Figure 1H*). The RCC was resected, followed by patch closure and aortic valve replacement. Histopathological tests showed severe calcification and fibrous changes in the resected aneurysm. As elastic fibres were preserved, the origin was considered the RCC valve leaflet (see Supplementary material online, *Image S2*; Victoria Blue staining). After the surgical procedure, the RVOT pressure gradient disappeared. On a 6-month follow-up, the patient remained asymptomatic and in good condition (see Supplementary material online, *S5* and *S6*).

Since he had no clinical history of suspected acquired SoV aneurysms, such as infection, trauma, or connective tissue disorders, we speculated that the RCC was sucked into a previously presumed ventricular septal defect (VSD) by the Venturi effect, and it led to the rare windsock deformation and RVOT obstruction.^1^ Firstly, this case highlighted that clinicians should note the importance of adhering to surgical indications with proper VSD follow-up, which potentially cause SoV aneurysm.^2^ Second, TTE is a convenient method for diagnosing SoV aneurysms. However, using only the TTE can be challenging for correct diagnosis, especially when coexisting with other heart defects.^3^ This case also highlighted the importance of employing multiple diagnostic modalities, including transoesophageal echocardiography, computed tomography, and magnetic resonance imaging, in rare cardiac conditions to achieve an accurate diagnosis.

## Supplementary Material

ytad559_Supplementary_Data

## Data Availability

No new data were generated or analysed in support of this research.
