# Supplementary figures and images for: A tongue-like Sinus of Valsalva Aneurysm with a pin-hole
Source: Eur Heart J Case Rep. 2023 Nov 9;7(11):ytad559. doi: 10.1093/ehjcr/ytad559 (PMC10686529; doi:10.1093/ehjcr/ytad559)

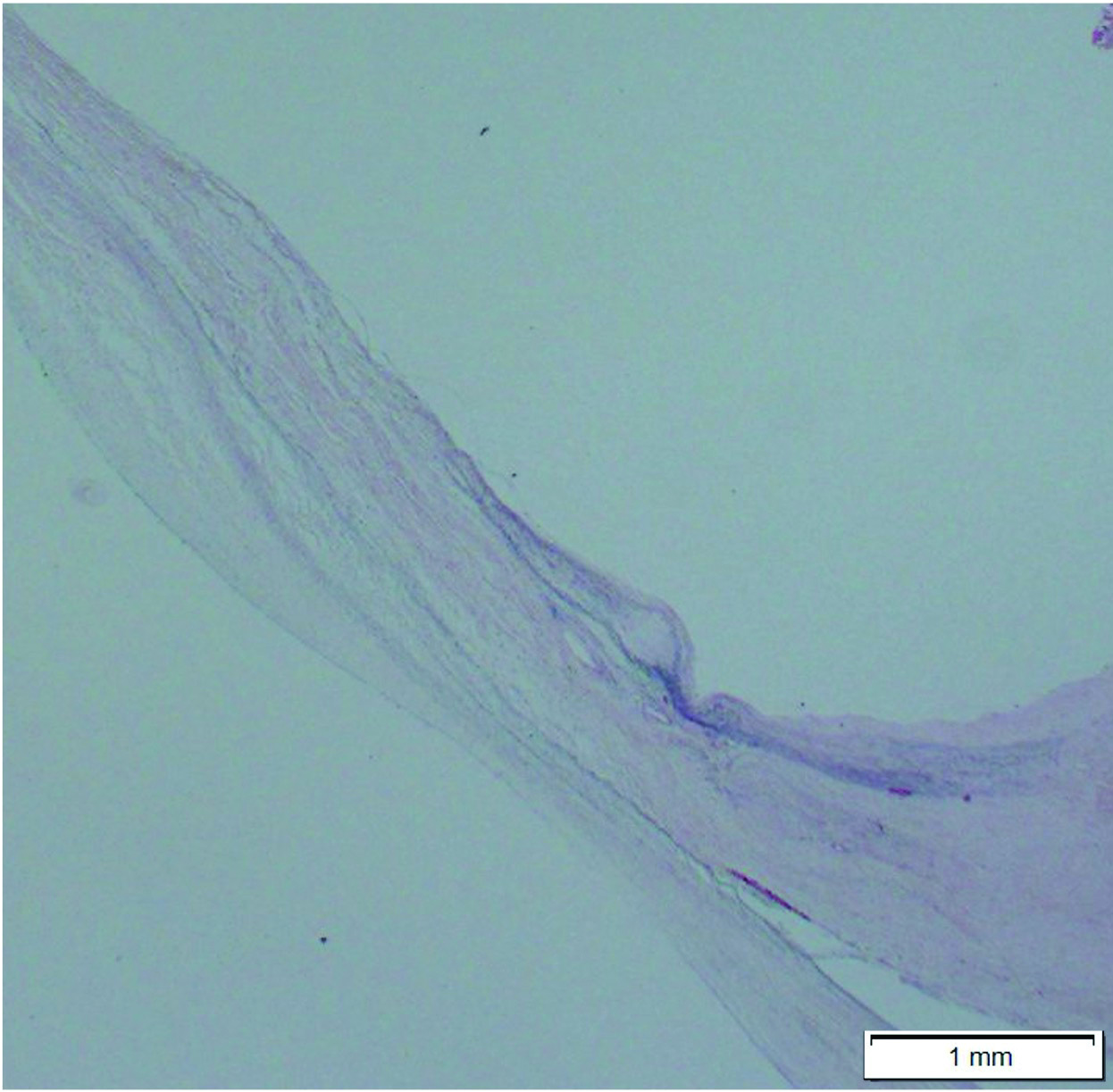

Supplement: ytad559_Supplementary_Data [file ytad559_supplementary_data.zip › Supplemantary image 2.jpg]

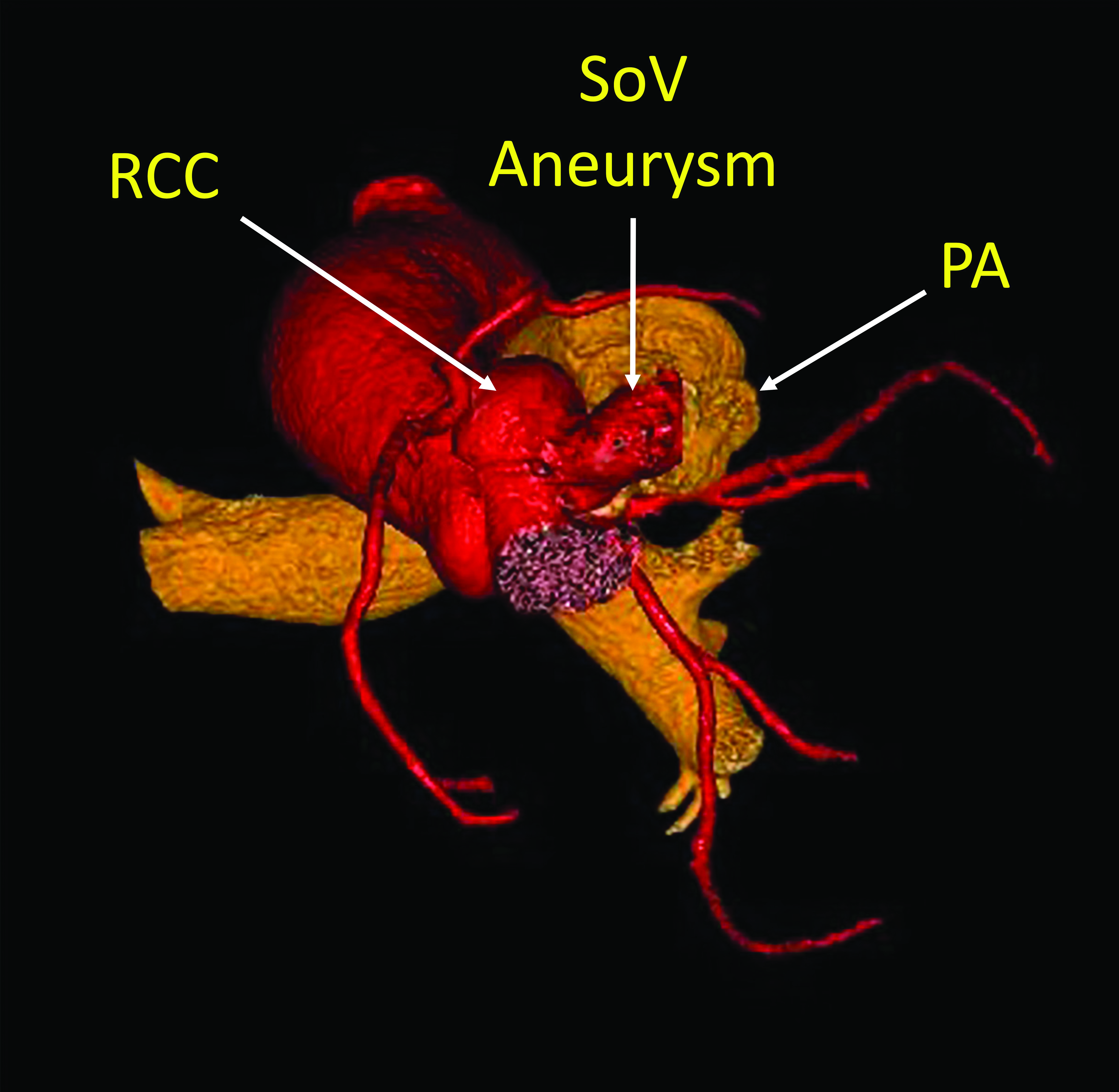

Supplement: ytad559_Supplementary_Data [file ytad559_supplementary_data.zip › Supplementary image 1.jpg]
